# m^5^C Regulator-Mediated Methylation Modification Patterns and Tumor Microenvironment Infiltration Characterization in Papillary Thyroid Carcinoma

**DOI:** 10.3389/fonc.2021.729887

**Published:** 2021-11-03

**Authors:** Fei Li, Qingmei Deng, Xiaoxi Pang, Shan Huang, Jingmiao Zhang, Xiaxia Zhu, Hong Chen, Xiuxia Liu

**Affiliations:** ^1^ Department of Nuclear Medicine, The Second Affiliated Hospital of Anhui Medical University, Anhui Medical University, Hefei, China; ^2^ Department of Molecular Pathology, Hefei Cancer Hospital, Chinese Academy of Sciences, Hefei, China

**Keywords:** papillary thyroid carcinoma (PTC), subtype, immune infiltration, 5-methylcytosine (m^5^C) modification, tumor microenvironment (TME)

## Abstract

Recently, immune response modulation at the epigenetic level is illustrated in studies, but the possible function of RNA 5-methylcytosine (m^5^C) modification in cell infiltration within the tumor microenvironment (TME) is still unclear. Three different m^5^C modification patterns were identified, and high differentiation degree was observed in the cell infiltration features within TME under the above three identified patterns. A low m^5^C-score, which was reflected in the activated immunity, predicted the relatively favorable prognostic outcome. A small amount of effective immune infiltration was seen in the high m^5^C-score subtype, indicating the dismal patient survival. Our study constructed a diagnostic model using the 10 signature genes highly related to the m^5^C-score, discovered that the model exhibited high diagnostic accuracy for PTC, and screened out five potential drugs for PTC based on this m5C-score model. m^5^C modification exerts an important part in forming the TME complexity and diversity. It is valuable to evaluate the m^5^C modification patterns in single tumors, so as to enhance our understanding towards the infiltration characterization in TME.

## Introduction

Thyroid cancer (THCA), a frequently-occurring endocrine cancer, takes up approximately 1.7% of human cancers (1). TC can be divided into subtypes, namely, alloplastic, follicular, medullary, and papillary thyroid cancer (PTC) (2). Of them, PTC shows the highest morbidity (75%–85% of thyroid cancers) (3). PTC can be cured under general conditions, and its survival rate at 5 years was over 95%, but PTC may sometimes differentiate to THCA, a malignancy with higher aggressiveness and mortality (4). Besides, approximately 30% of PTC cases suffer from tumor recurrence (5). As a result, analyzing the disease features at the molecular level is essential.

It is increasingly suggested that RNA modification at the post-transcriptional level exerts a vital part in a variety of malignancies (6, 7). RNA and histone alterations at epigenetic and genetic levels are extensively investigated in the context of tumor progression; as a result, numerous therapeutic means have been developed, such as the drugs that target the hypoxic pathways and the histone deacetylase inhibitors (8). In the living body, over 150 RNA modifications are modified as the third epigenetics layer, such as N1-methyladenosine (m^1^A) and N6-methyladenosine (m^6^A), together with 5-methylcytosine (m^5^C) (9–13).

Of them, m^5^C modification, a reversible RNA post-transcriptional modification, exerts an important part in the regulation of mRNA translation, export, alternative splicing (AS) and stabilization localization (14, 15). m^5^C in mRNAs has been extensively studied, and many articles reveal that m^5^C greatly affects mRNAs, tRNAs, and rRNAs (16). The m^5^C methylation is related to various regulators, such as the m^5^C “readers”, demethylases, and methyltransferases. Typically, the methyltransferase “writer” complex enhances RNA methylation at the C5 position, whereas the distinct “reader” proteins are responsible for recognizing and binding to methylated mRNAs, and “eraser” protein is in charge of reversing the m^5^C modification through the degradation of written methylation. The adenosine demethylases, methyltransferases, together with the RNA-binding proteins involved in m^5^C modification are referred to as the m^5^C “erasers” (like TET2), m^5^C “writers” (like NSUN1-7, DNMT1-2, and DNMT3A-3B), as well as m^5^C “readers” (like ALYREF) (17). More and more studies suggest that m^5^C modification exerts an important part in a variety of critical pathophysiological processes, such as the dysregulated cell proliferation and death, abnormal immune modulation, developmental defects, malignant development of tumor, and damaged self-renewal ability (18–20). Nonetheless, the typical gene signatures, together with the diagnostic and prognostic significance of m^5^C-related regulators in PTC, are still unclear.

Immunotherapy based on the immunological checkpoint inhibitors (PD-1/L1, ICB, or CTLA-4) is found to be effective on certain patients who have persistent responses. However, most patients can only gain small or even no benefit from immunotherapy (21). In traditional practice, tumor progression is recognized to be the multi-step process involving variations within tumor cells at epigenetic and genetic levels, but many articles reveal that the tumor microenvironment (TME) for the development and survival of tumor cells also exerts an important part during tumor progression (22). There is a complicated TME in tumor, which contains tumor cells and stromal cells like macrophages and resident fibroblasts [cancer-associated fibroblast (CAF)]. In addition, it also contains distant recruited cells like the infiltrating immunocytes (lymphocytes and myeloid cells), bone marrow-derived cells (BMDCs) like hematopoietic and endothelial progenitor cells, the secretory factors (like chemokines, cytokines, and growth factors), and new vessels (23). Notably, the tumor-associated myeloid cells (TAMCs) are composed of five different myeloid subsets, namely, myeloid-derived suppressor cells (MDSCs), tumor-associated macrophages (TAMs), tumor-associated neutrophils (TANs), Tie2-expressing monocytes, and dendritic cells (DCs) (24). Tumor cells can trigger changes in biological behaviors *via* directly or indirectly interacting with other components in the TME; for instance, the induction of new vessel formation and proliferation, apoptosis inhibition, hypoxia prevention, and immune tolerance induction (25). The TME complexity and diversity have been increasingly revealed, and TME is found to play an important part in immune escape and tumor progression, together with its impact on immunotherapy response (26, 27). It is critical to predict ICB response according to TME cell infiltration characterization, so as to increase the success rate of current ICBs and to exploit the new immunotherapies. Consequently, the comprehensive analysis of the complexity and diversity of TME landscapes helps to identify the diverse tumor immune phenotypes and to guide and predict responses to immunotherapies (28). Furthermore, it also contributes to revealing the potential biomarkers, thus facilitating to recognize the immunotherapy responses in patients and develop the novel therapeutic targets (29).

Individual recent articles suggest that the TME infiltrating immunocytes are related to m^5^C modification, and such relationship cannot be interpreted through the mechanism of RNA degradation (30, 31). Nonetheless, these articles only focus on holistic 5-hydroxymethylcytosine (5hmC) levels or cell types because of the technical restrictions, and the anticancer efficacy is evaluated based on a number of the highly coordinated tumor suppressor factors. Consequently, it is necessary to comprehensively recognize cell infiltration features within TME under the regulation of several m^5^C regulators, so as to shed more light on the TME immunomodulation. The present work combined genome data from 493 TCGA-PTC samples for the comprehensive evaluation of m^5^C modification patterns, and related them to cell infiltration features within TME. Altogether, three different m^5^C modification patterns were identified, under which the high differentiation degree of TME features were found, indicating the critical part of m^5^C modification in forming individual TME features. On this basis, the scoring system was also established for the quantification of m^5^C modification patterns for individual cases. Finally, this study mined the m^5^C-score-related signature genes to construct the PTC diagnostic model using the support vector machine (SVM) method.

## Materials and Methods

### Source and Preprocessing of PTC Data

The work flow chart in the present work is presented in **Supplementary Figure 1**. The Cancer Genome Atlas (TCGA) and the Gene Expression Omnibus (GEO) databases were searched to obtain the clinical annotation and related gene expression data. Patients who had no survival data were eliminated from this study. The eligible PTC cohorts [including GSE33630 (32), GSE65144 (33), and GSE29265, together with TCGA-PTC (The Cancer Genome Atlas- papillary thyroid carcinoma)] were collected into the present work. With regard to Affymetrix^®^ microarray data, raw “CEL” files were downloaded to adjust the background and normalize the quantile using the multiarray averaging approach by affy and simpleaffy packages. In terms of microarray data of additional sources, matrix files after normalization were collected directly. For TCGA datasets, the RNA sequencing information (FPKM values) of gene levels was obtained based on the Genomic Data Commons (GDC, https://portal.gdc.cancer.gov/) by TCGAbiolinks of R package, a software designed to comprehensively analyze GDC data (34). Thereafter, the FPKM values were converted to the transcripts per kilobase million (TPM) values. At the same time, the GSE65144 (12 tumor and 13 normal samples), GSE33630 (60 tumor along with 45 normal samples), and GSE29265 (29 tumor and 20 normal samples) datasets were also downloaded. R package (version 3.6.1) was utilized for data analysis.

### Consensus Clustering of the 13 m^5^C Regulators

Altogether, 13 regulators were obtained based on TCGA datasets to identify the diverse m^5^C regulator-mediated m^5^C modification patterns. All the 13 genes, except for ALYREF and NSUN1, were with available expression profiles. The remaining 11 m^5^C regulators contained one eraser (TET2) and 10 writers (NSUN2-7, DNMT1-2, and DNMT3A-3B). Of our 493 patients from TCGA-PTC, 9 among those 11 genes were differentially expressed between tumor and normal tissues (with the exception for NSUN3 and DNMT3A) (**Supplementary Table 1** and **Supplementary Figure 2**). Later, consensus clustering was adopted for identifying the different m^5^C modification patterns according to nine m^5^C regulator expression levels, and then patients were classified accordingly. The above procedure was performed using the ConsensuClusterPlus package (35) for 1000 iterations to guarantee the classification stability.

### Gene Set Variation Analysis together With Functional Annotation

To investigate the heterogeneities in biological process among the m^5^C modification patterns, GSVA was carried out by the “GSVA” R package. Notably, GSVA is the unsupervised, non-parametric approach usually used to estimate variations of pathways and biological processes within the expression dataset samples (36). The “c2.cp.kegg.v7.0.symbols” gene sets were extracted based on MSigDB database to conduct GSVA. The adjusted *p* < 0.05 indicated statistical significance. Meanwhile, functional annotation was performed using WebGestaltR package (37), and the threshold was FDR < 0.05.

### TME Cell Infiltration Estimation

Estimate R package was utilized to calculate immune and stromal scores for all samples to reflect the immune and stromal cell infiltration degrees on the whole. Besides, CIBERSORT algorithm (38) was adopted for quantifying cell infiltration relative abundance within the TME of PTC. Thereafter, the gene set used to mark the TME-infiltrating immunocyte type was acquired to score different human immunocyte subtypes, like the activated CD8 T cells, regulatory T cells, natural killer T cells, activated dendritic cells (DCs), and macrophages (39).

### Discovery of Differentially Expressed Genes Across the Different m^5^C Phenotypes

To identify the m^5^C-associated genes, the patients were divided into three different m^5^C modification patterns according to nine m^5^C regulator expression levels. DEGs were determined across the diverse modification patterns using the empirical Bayesian method in the limma R package (40). The adjusted *p* < 0.05 served as the significance criterion to determine DEGS.

### m^5^C Gene Signature Construction

To quantify m^5^C modification patterns among individual tumors, the scoring system, m^5^C-score, was built based on the m^5^C gene signature, as shown below:

First of all, DEGs obtained based on the diverse m^5^C-clusters were subjected to normalization across all samples, and then, those overlapped genes were selected. Afterwards, all cases were divided into different groups *via* the unsupervised clustering approach, so as to analyze the overlapped DEGs. In addition, the gene cluster number and the stability were defined using the consensus clustering algorithm. Later, prognostic analysis was carried out for all genes selected in our constructed signature by the use of the univariate Cox regression model. Later, those significant genes were obtained in subsequent analysis. In this study, *p* < 0.01 was selected as the criterion to screen 49 genes. **Supplementary Table 2** shows the results of single factor survival analysis for the 49 genes. Then, principal component analysis (PCA) was utilized for constructing the m^5^C signature. PC1 and PC2 were adopted as the signature scores; as a result, the score was focused on the set that had the greatest number of well-correlated (or anticorrelated) genes, and the contributions of genes not tracking with other members in the set were down-weighted. Later, the m^5^C-score was defined by the GGI-like approach (41):

${\text{m}}^{5}\text{C}-\text{score }=\;\sum \left(\text{PC}1_{\text{i}}+\text{ PC}2\text{i}\right)$

In the formula, i represents the 49 m^5^C phenotype-associated gene expression levels.

### m^5^C-Score Based PTC Diagnostic Model Establishment

First of all, this study mined the signature genes significantly correlated with the m^5^C-score (correlation coefficient > 0.4), and the PTC diagnostic model was constructed by the SVM method. Thereafter, the accuracy of this model was verified using samples from TCGA and GEO databases.

### Statistical Methods

The Spearman and distance correlation analysis was adopted to calculate the correlation coefficients of TME-infiltrating immunocytes with m^5^C regulator expression levels. Thereafter, Kruskal–Wallis test and one-way ANOVA were applied in comparing the heterogeneities among three groups. Based on correlations of the m^5^C-score with patient survival, the survminer R package was utilized to determine the threshold value for every dataset. In addition, the “surv-cutpoint” function was used for dichotomizing the m^5^C-score by testing the possible threshold values to find the maximal rank statistic. Later, all cases were classified as high or low m^5^C-score group according to the maximal log-rank statistics for reducing the calculation batch effect. In the meantime, the log-rank test and Kaplan–Meier approach were adopted for identifying the significance of differences, so as to generate survival curves. The univariate Cox regression model was used for calculating hazard ratios (HRs) for the m^5^C regulators as well as the m^5^C phenotype-associated genes. Meanwhile, the receiver operating characteristic (ROC) curve was plotted to assess the sensitivity and specificity of our diagnostic model and the m^5^C-score, and pROC R package was utilized to quantify the area under the curve (AUC). The two-sided statistical *p* < 0.05 indicated statistical significance. The R 3.6.1 software was employed for data processing.

## Results

### The Nine-Regulator-Mediated m^5^C Methylation Modification Patterns

**Figure 1:**
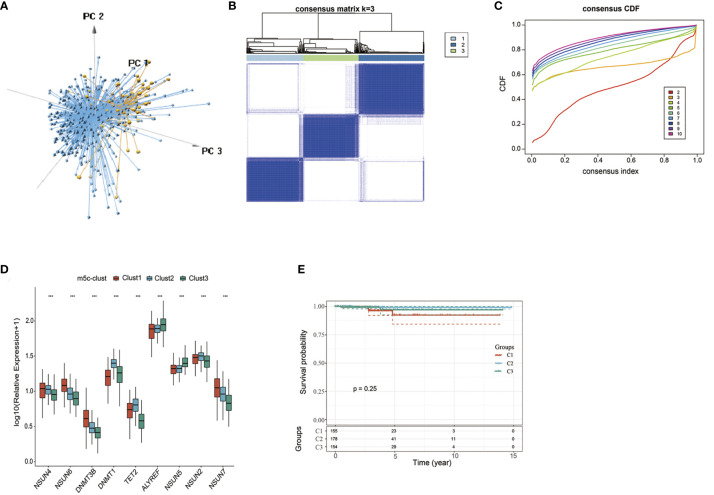
The nine-regulator-mediated m^5^C methylation modification patterns. **(A)** PCA analysis based on nine m^5^C regulators. **(B, C)** Consensus clustering was adopted for identifying the different m^5^C modification patterns. **(D)** The expression levels of 9 m^5^C regulators in different m^5^C modification patterns. **(E)** Survival analysis of the three subtypes. ***statistical significance.

According to nine m^5^C regulators with expression profiles in the TCGA-PTC dataset, PTC samples were identified from normal samples (**Figure 1A**). Afterwards, the expression profile data of these nine m^5^C regulators were carried out with *z*-score standardization using the scale function in mosaic package. Then, three different m^5^C modification patterns were discovered according to those nine m^5^C regulator expression patterns (**Figures 1B, C**). These three patterns were named m^5^C-clusters 1–3. It was observed from **Figure 1D** that the expression level of these nine m^5^C regulators showed significant differences among the three distinct subtype samples.

Prognostic analysis was also carried out for these three major m^5^C modification patterns, which suggested that the m^5^C-cluster 2 modification pattern showed survival advantage (**Figure 1E**). However, due to the speciality of PTC and the good overall prognosis, there was no significant statistical difference among these three subtypes. Besides, average survival time of samples in these three subtypes was also analyzed, which discovered that the average survival time of C2 subtype samples was 1307.657 days, that of C1 subtype samples was 1125.877 days, and that of C3 subtype samples was 1202.695, with that in C2 higher than those in C1 and C3.

### TME-Infiltrating Cell Features in Different m^5^C Modification Patterns

**Figure 2:**
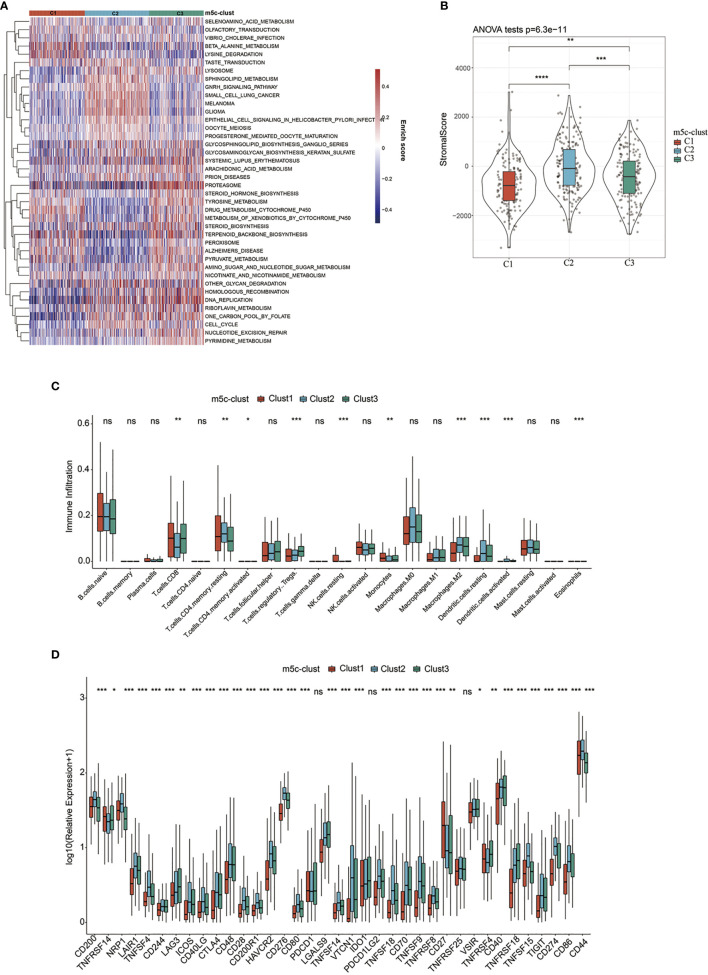
TME-infiltrating cell features in different m^5^C modification patterns. **(A)** Biological behaviors in the different m^5^C modification patterns were conducted by GSVA. **(B)** Levels of stromal scores in different m^5^C modification patterns. **(C)** The levels of infiltration of 22 immune cells in different m^5^C modification patterns. **(D)** The expression levels of 37 immune checkpoints in different m^5^C modification patterns. *, **, ***, **** statistic difference at different levels; ns, no significance.

To explore those biological behaviors in the different m^5^C modification patterns, GSVA was conducted. It was illustrated from **Figure 2A** that m^5^C-cluster 1 significantly associated with the amino acid metabolic pathways; m^5^C-cluster 2 was enriched to the endocrine system, lipid metabolism, and cancer, whereas m^5^C-cluster 3 was associated with cell cycle, DNA repair, and nucleic acid metabolism.

**Figure 3:**
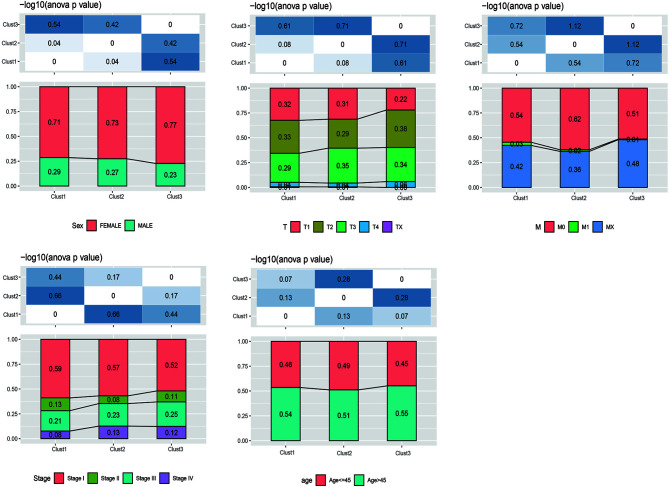
The distribution of clinical features (gender, stage, and age) of samples in the m^5^C-cluster 1–3.

Furthermore, the distribution of clinical features of samples in the above three subtypes was statistically analyzed. The statistical results are displayed in **Supplementary Table 3** and **Figure 3**. It was found from the results that, multiple clinical features in the three subtype samples were randomly distributed, with no significant difference.

In addition, the ESTIMATE algorithm was applied in quantifying the differences in stromal cell infiltration among the three subtype samples. As shown in **Figure 2B**, the stromal score in m^5^C-cluster 2 was the highest, followed by m^5^C-cluster 3, while m^5^C-cluster 1 had the lowest score. In addition, there were significant differences among them. Thereafter, the CIBERSORT deconvolution algorithm was utilized for comparing the heterogeneities in immunocyte components of the three m^5^C modification patterns (**Figure 2C**). Meanwhile, the support vector regression was used to determine the immunocyte types in tumors. As a result, high levels of Tregs and monocytes were detected in m^5^C-cluster 1 and m^5^C-cluster 3, whereas excessive resting/activated DCs were found in m^5^C-cluster 2. Recently, research has particularly focused on the RNA modification mechanism in the regulation of DC activation. DCs function to present antigen and to activate the naive T cells, which connect the intrinsic immunity with the adaptive one (42).

Finally, this study analyzed the expression of the 34 known immune checkpoints in the three subtype samples. As found from **Figure 2D**, there were significant differences in the expression of these 34 immune checkpoints among the three subtypes. Most immune checkpoint genes were highly expressed in m^5^C-cluster 2, followed by m^5^C-cluster 3, while m^5^C-cluster 1 had the lowest expression, which was consistent with the average survival time of samples in the three subtypes.

### m^5^C Gene Signature Establishment Along With Functional Annotation

**Figure 4:**
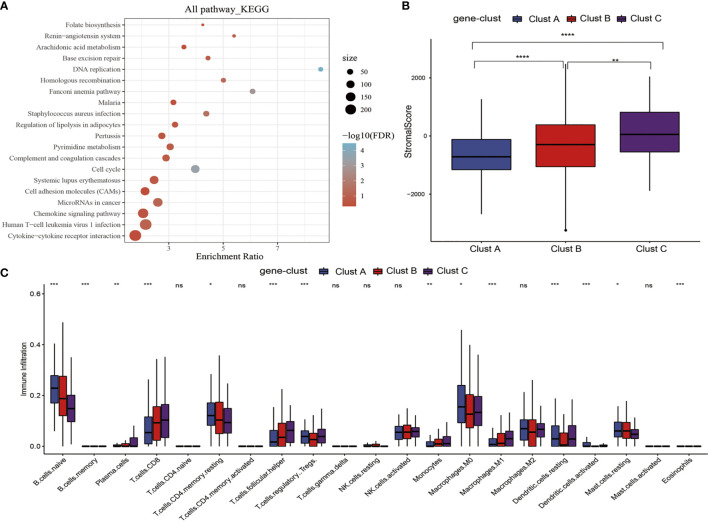
m^5^C gene signature establishment along with functional annotation. **(A)** KEGG enrichment analysis of 690 DEGs. **(B)** Levels of stromal scores in three m^5^C gene-cluster subtypes. **(C)** The levels of infiltration of 22 immune cells in three m^5^C gene-cluster subtypes. *, **, ***, **** statistic difference at different levels; ns, no significance.

To better investigate the possible biological behaviors of all the m^5^C modification patterns, the limma package was used to determine 690 m^5^C phenotype-associated DEGs (**Supplementary Figure 3**). In addition, KEGG pathway enrichment analysis was carried out on DEGs using the WebGestaltR package. It was surprising that these genes were enriched to cell cycle, DNA repair, cell adhesion molecules, and immune inflammatory response-related pathways. These findings verified the important role of m^5^C modification in cancer cells themselves and in TME immunomodulation (**Figure 4A**). To better validate such regulatory mechanism, the unsupervised clustering analysis was performed using those 690 m^5^C phenotype-associated genes, for the sake of classifying cases to distinct genome subtypes. Similar to clustering analysis of m^5^C modification patterns, three different m^5^C modification genome phenotypes were found, which were referred to as m^5^C gene-clusters A–C, separately (**Supplementary Figure 4**). According to such results, there were three different m^5^C methylation modification patterns in PTC. Besides, there were diverse signature genes in the three different gene clusters (**Supplementary Figure 4**). The m^5^C regulator expression levels were significantly different among the three m^5^C gene-clusters (**Supplementary Figure 5**), consistent with the results obtained for m^5^C methylation modification patterns. The expression quantities of these nine genes were the highest in gene-cluster B samples, followed by gene-cluster A samples, and were the lowest in the gene-cluster C samples.

### Clinical Features and Transcriptome Traits of the m^5^C-Associated Phenotypes

First of all, we analyzed the stromal scores of three m^5^C gene-cluster subtypes. The results suggested that (**Figure 4B**) there were significant differences in the stromal score of three subtypes, among which, gene-cluster C had the highest score, followed by gene-cluster B, while gene-cluster A had the lowest score. Then, we analyzed the distribution of 22 immunocytes in the three m^5^C gene-cluster subtypes. As observed from **Figure 4C**, the distribution of 15 immunocytes in three subtypes showed statistically significant differences. These findings revealed the important role of m^5^C methylation modification in the formation of diverse TME landscapes and tumor-related immune regulation.

**Figure 5:**
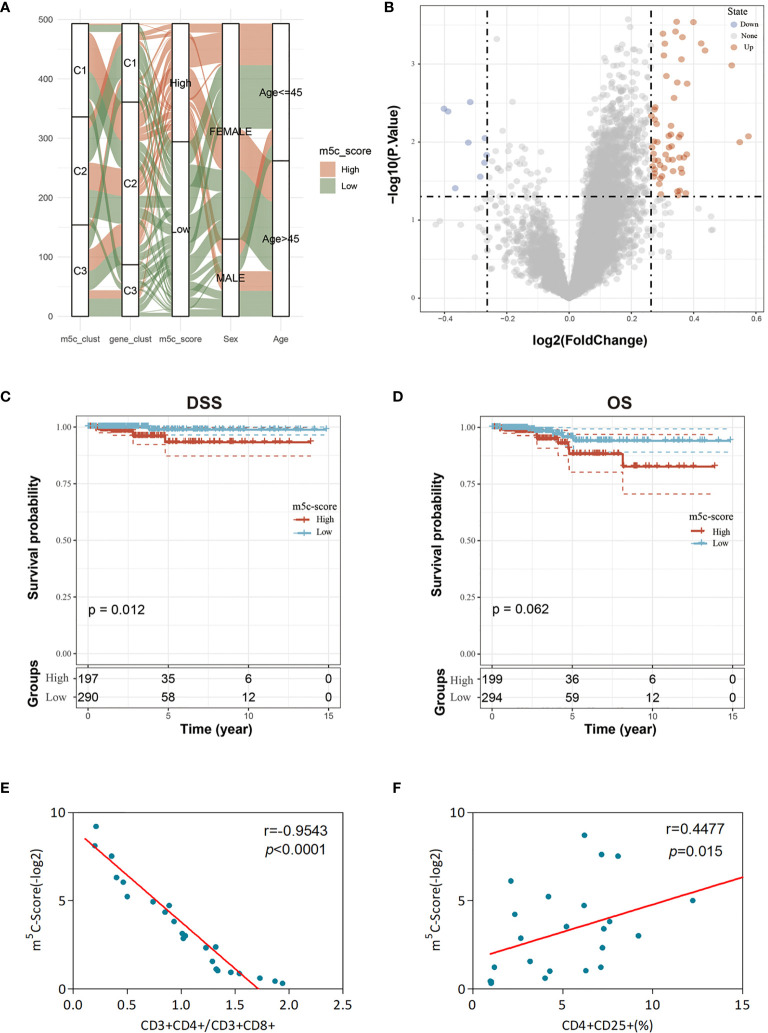
Clinical features and transcriptome traits of the m^5^C-associated phenotypes. **(A)** Alluvial diagram showing the changes of m^5^C modification patterns, gender, age, gene cluster, and the m^5^C-score. **(B)** DEGs between high and low m^5^C-score samples. **(C, D)** Differences in DFS **(C)** and OS **(D)** between high and low m^5^C-score samples. **(E)** Relationship between the m^5^C-score value and the score of CD3+CD4+/CD3+CD8+ cells of the peripheral blood samples of 24 GBM patients. The m^5^C-Score value was negatively associated with the ratio of CD3+CD4+/CD3+CD8+ cells. **(F)** Relationship between the m^5^C-score value and the percentage of CD4+CD25+ Tregs in peripheral blood samples of the 24 GBM patients. The m^5^C-score value was positively related with the percentage of CD4+CD25+ Tregs.

Nonetheless, the above results were obtained from patient population alone, which might not precisely estimate the m^5^C methylation modification patterns of individual cases. Due to the m^5^C modification complexity and heterogeneity in individual samples, this study established the scoring system (m^5^C-score) using the phenotype-associated genes for quantifying m^5^C modification patterns in individual PTC cases. Besides, those attribute alterations in individual patients were visualized by the alluvial diagram (**Figure 5A**). It was discovered from the figure that, among the 3 m^5^C-cluster subtypes, samples in m^5^C-cluster 2 and m^5^C-cluster 3 subtypes were mostly distributed in the low m^5^C-score group, while those in the high m^5^C-score group were basically derived from the m^5^C-cluster 1 subtype. In the three m^5^C gene-cluster subtypes, the m^5^C-score values of Cluster A and Cluster C samples were lower. Samples aged over 40 years were mostly classified into the low m^5^C-score group, while females mostly belonged to the high m^5^C-score group.

To further evaluate the differences between low and high score samples, the limma package was used to analyze the DEGs between the two groups. Using the thresholds of logFC > log_2_(1.2) and *p* < 0.05, 67 DEGs were screened, including 58 upregulated and 9 downregulated ones (**Figure 5B**). Moreover, the WebGestaltR package was utilized for the GO and KEGG enrichment analyses of DEGs, with *p* < 0.05 as the threshold. A total of 62 biological processes (BP), 2 cellular components (CC), 6 molecular functions (MF), and 9 pathways were selected. As shown in **Supplementary Figure 6**, these genes were mainly involved in tumor proliferation and immune response-related biological processes/molecular functions and signaling pathways, such as MAPK, TNF, and IL-17.

Subsequently, this study observed the correlation of the m^5^C-score with patient survival and analyzed the difference in prognosis between high and low m^5^C-score samples. The results suggested that, samples with low m^5^C-scores had better prognosis than those with a high score, regardless of DFS or OS (**Figures 5C, D**). In addition, it was also discovered that there was no difference in the clinical features (such as T, M, and stage) between high and low m^5^C-score samples (**Supplementary Figure 7**). The expression levels of nine m^5^C regulators in the high m^5^C-score group were significantly higher than those in the low score group, and there was significant difference between the two groups (**Supplementary Figure 8**).

Subsequently, this study observed the correlation of the m^5^C-score with TME. First of all, the CIBERSORT method was adopted to evaluate the infiltration level of each immunocyte type in the high and low m^5^C-score TCGA-TPC samples. The results are presented in **Supplementary Figure 9A**. There were significant differences in six cell types between high and low m^5^C-score groups. In addition, this study also calculated the stromal score, immune score, and ESTIMATE score in different samples. As presented in **Supplementary Figure 9B**, in the low m^5^C-score group, the immune score was significantly higher than that in the high m^5^C-score group, which was consistent with the previous results that show that the low m^5^C-score group had better prognosis than the high score group. Moreover, it was discovered through expression of immune checkpoint genes that there were significant differences in 16 immune checkpoint gene expression levels between high and low m^5^C-score groups (**Supplementary Figure S10**). Based on these findings, low m^5^C-score showed close correlation with immune activation. Furthermore, the m^5^C-score helped to assess m^5^C modification patterns in individual tumors and better assess the TME cell infiltration features of tumors, thus contributing to distinguishing the true or false TME immune infiltration.

**Figure 6:**
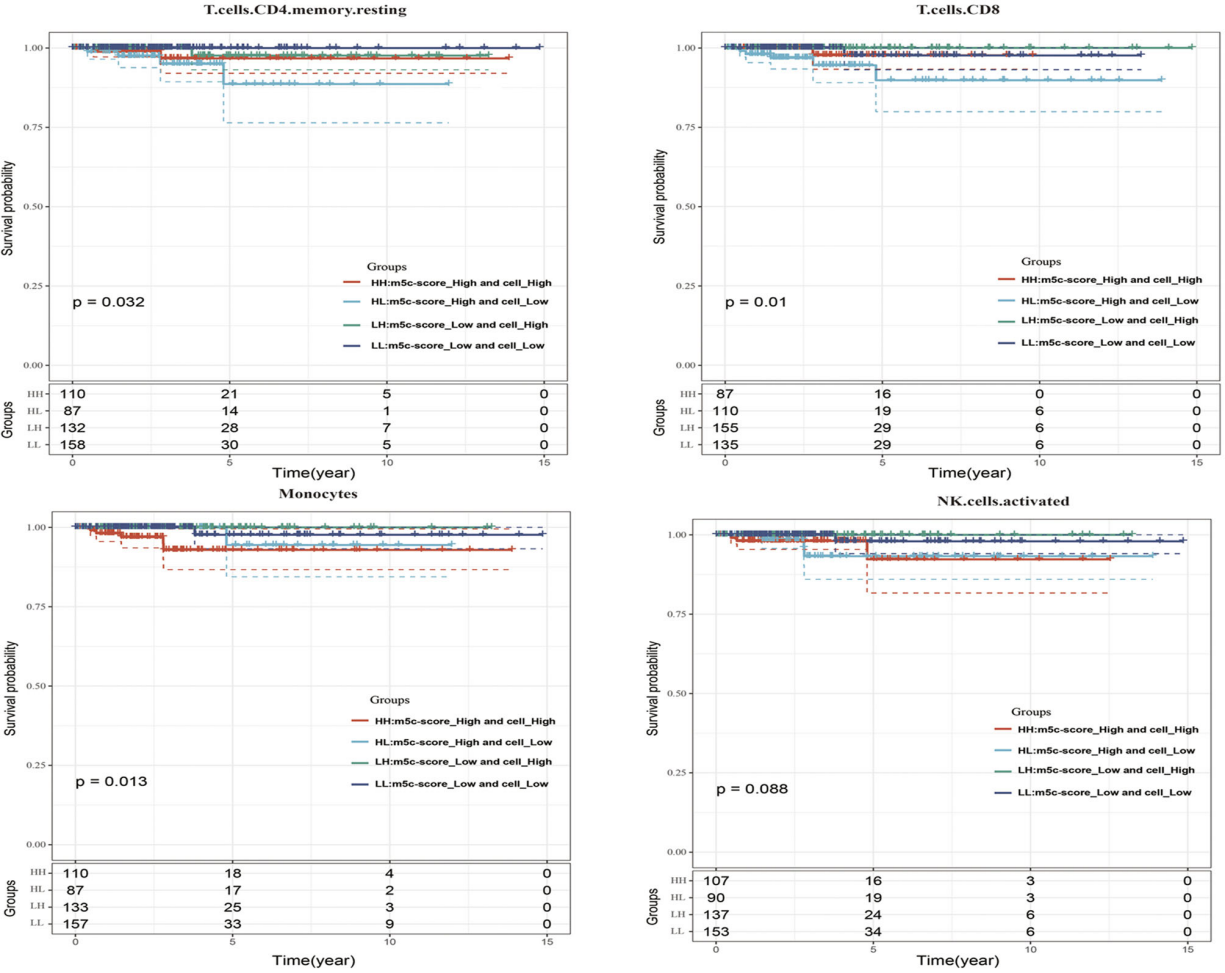
The influences of the m^5^C-score and various immunocyte (resting CD4^+^ memory T cells, CD8^+^ T cells, activated NK cells, and monocytes) infiltration levels on the prognosis for PTC patients.

Last, this study integrated the influences of the m^5^C-score and various immunocyte infiltration levels on the prognosis for PTC patients. From **Figure 6**, it was discovered that resting CD4^+^ memory T cells and CD8^+^ T cells were mainly enriched in low m^5^C-score samples, while activated NK cells and monocytes were mostly enriched in the high m^5^C-score group. Then, the median infiltration level of the above four cell types was used to divide all samples into high and low immunocyte infiltration level groups. It was discovered that samples with low m^5^C-score and low infiltration level of resting CD4^+^ memory T cells had the best prognosis, while those with high m^5^C-score and low infiltration level of resting CD4^+^ memory T cells had the poorest prognosis. In addition, samples with low m^5^C-score and high CD8^+^ T cell infiltration had the best prognosis, while those with high m^5^C-score and low CD8^+^ T cell infiltration had the poorest prognosis. Furthermore, it was found that samples with low m^5^C-score and high monocyte infiltration had the best prognosis, while those with high m^5^C-score and high monocyte infiltration had the poorest prognosis. According to the prognostic prediction model, we analyzed the correlation between the m5C-score and Treg expression in 24 PTC patients. The m5c-score showed a negative relationship with CD3+CD4+/CD3+CD8+ (*r* = −0.9543, *p* < 0.0001; **Figure 5E**), but a positive relationship with CD4+CD25+ Tregs percentage (*r* = 0.4477, *p* = 0.015; **Figure 5F**).

### Construction and Verification of the m^5^C-Score-Based PTC Diagnostic Model

First of all, this study calculated the correlation of 49 m^5^C phenotype-related genes with the m^5^C-score. Then, 10 signature genes related to the m^5^C-score were screened by the threshold of correlation coefficient >0.4, which were used as the features to construct the SVM classification model.

**Figure 7:**
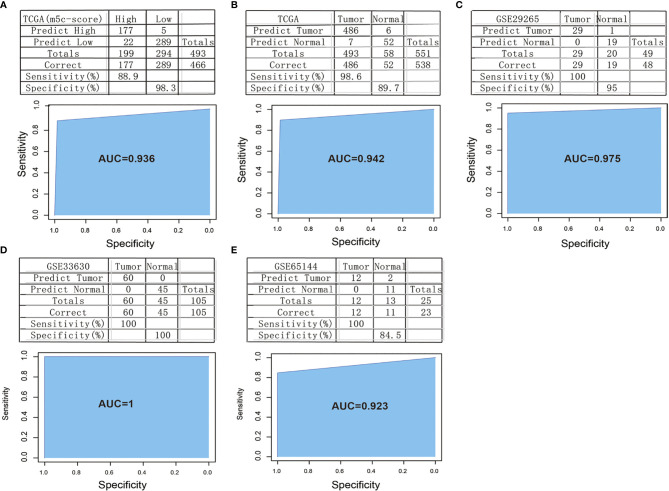
Construction and verification of the m^5^C-score-based PTC diagnostic model. **(A)** Comparison of classification results of TCGA-PTC samples by diagnostic model constructed based on 10 signature genes and the m^5^C-score. **(B)** Accuracy of classification of TCGA samples by a diagnostic model constructed based on 10 signature genes. **(C–E)** Accuracy of classification of samples in GSE29265 **(C)**, GSE33630 **(D)**, and GSE65144 **(E)** by a diagnostic model constructed based on 10 signature genes.

In order to verify the classification efficiency and accuracy of the model, we used the expression profile data of TCGA tumor samples as the training set. The m^5^C-score was utilized to classify the samples into high and low groups. Then, the expression profile data of these 10 genes were used to construct the SVM classification model to classify the TCGA-TPC samples. It was discovered that, compared with the m^5^C-score classification results, the accuracy reached 98.3%, and the sensitivity was up to 88.9%. The 493 samples were accurately classified, with an area under the ROC curve (AUC) of 0.936 (**Figure 7A**). The above results demonstrated that the classification model constructed based on these 10 signature genes well simulated the classification results of the m^5^C-score. The gene number was substantially reduced, which significantly improved the classification efficiency.

Thereafter, all the 551 TCGA samples (including 493 tumor samples and 58 normal samples) were used as the verification set 1. The abovementioned 10 genes were used as the features to construct the SVM classification model to classify the samples. Surprisingly, it was discovered that the model accurately classified TCGA-TPC samples into tumor samples and para-carcinoma tissue samples, with a classification accuracy of 89.7% and a sensitivity of 98.6%. Of the 551 samples in verification set 1, 538 were accurately classified, with an AUC of 94.2% (**Figure 7B**).

To further verify the model classification efficiency and accuracy, another three sets of microarray data were also downloaded, and the 10 signature genes were used for SVM verification. The GSE29265 dataset was utilized as verification set 2, which included 49 samples (20 normal samples and 29 tumor samples), with a model classification accuracy of 95%. Of the 49 samples, 48 were accurately classified, the model sensitivity to high and low scores was up to 100%, and the AUC was 97.5% (**Figure 7C**). Meanwhile, the GSE33630 dataset was used as verification set 3, which included 105 samples (45 normal samples and 60 tumor samples). The model classification accuracy reached up to 100%, all the 105 samples were accurately classified, the model sensitivity to high and low scores was 100%, and the AUC was 100 (**Figure 7D**). The GSE65144 dataset was used as verification set 4, which contained 25 samples (13 normal samples and 12 tumor samples). The model classification accuracy was 84.5%, all the 25 samples were accurately classified, the model sensitivity to high and low scores was 100%, and the AUC was 92.3% (**Figure 7E**).

### Potential Drug Screening and Evaluation for the m5C-Score-Based PTC Diagnostic Model

**Figure 8:**
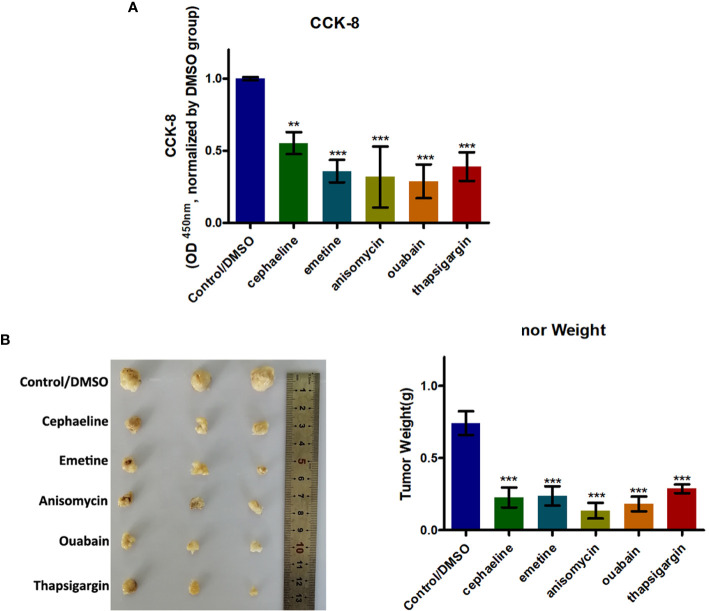
Five potential drugs based on the PTC m5C-score model could impair growth of PTC cells. **(A)** Histogram showing the viability of PTC cells with or without five potential drugs (cephaeline, emetine, anisomycin, ouabain, and thapsigargin) for 48 h at 20 μM. **(B)** BALB/c mice were subcutaneously injected with PTC cells. After 5 days, the nude mice were treated with cephaeline, emetine, anisomycin, ouabain, or thapsigargin (100 mg/kg daily, intraperitoneal injection). Tumor weights were measured after 6 weeks (*n* = 5 mice/group). ***statistical significance.

We firstly used the L1000 fireworks display (L1000FWD) tool, and a reverse drug screening method for deferentially expressed genes in high- and low-risk groups of the m^5^C-score and obtained small molecules (drugs, **Supplementary Table 4**). In the interaction database between CMAP drug and gene expression, we analyzed 67 drugs that may interact with genes with different changes in the risk model constructed by the m^5^C-score, and selected 55 small molecules (drugs, **Supplementary Table 5**). We compared the potential drug overlap between L1000 and CMAP annotation, and found that there were five overlapping small molecules (S8), namely cephaeline, emetine, anisomycin, ouabain, and thapsigargin. CCK8 was used to detect the effect of five potential drugs on the growth and metabolic activity of PTC tumor cells. It was found that compared with the control group, the five drugs could inhibit the growth of thyroid cancer cells in different degrees (**Figure 8A**). Consistent with this, results of subcutaneous transplantation model also showed that intraperitoneal injection of these five drugs could significantly inhibit the growth of tumor, respectively (**Figure 8B**).

## Discussion

More and more studies suggest that the m^5^C modification interacts with different m^5^C regulators to play a vital part in anticancer efficacy, inflammation, and intrinsic immunity. A majority of articles have focused on the individual TME cell type or individual regulator, yet no study has completely identified the TME infiltration features mediated by several m^5^C regulators simultaneously. It is important to identify the different m^5^C modification patterns within the TME-infiltrating cells, so as to display the anticancer immune response in TME and to guide the efficient immunotherapies.

In this study, on the basis of those nine m5C regulators, three different m^5^C methylation modification patterns were identified, which showed different TME-infiltrating features. Furthermore, differences in mRNA transcriptome data across different m^5^C modification patterns were suggested to be remarkably related to the biological pathways associated with m^5^C and immunity. Such DEGs were recognized to be the m^5^C-associated signature genes. Consistent with the m^5^C modification phenotype clustering analysis results, three genomic subtypes were found using the m^5^C signature genes, and they showed significant correlations with the immune and stromal activation. According to such results, m^5^C modification played an important role in the formation of diverse TME landscapes. As a result, comprehensively assessing m^5^C modification patterns can shed more light on the features of TME cell infiltration. Due to the differences in individual m^5^C modification patterns, quantifying m^5^C modification patterns in individual tumors is necessary. To this end, the scoring system, namely, the m^5^C-score, was constructed in the present work for evaluating m^5^C modification patterns in PTC cases. Our results show the reliability and robustness of the m^5^C-score to comprehensively assess the m^5^C modification patterns of individual tumors, and it might be used to better examine TME infiltration patterns (namely, the immune phenotypes of tumor). Integrative analysis further revealed that the m^5^C-score might serve as a biomarker to independently predict the PTC prognosis. Finally, this study constructed a diagnostic model using the 10 signature genes highly related to the m^5^C-score and discovered that the model exhibited high diagnostic accuracy for PTC.

The m^5^C-score might be adopted clinically for the comprehensive evaluation of m^5^C methylation modification patterns together with related TME cell infiltration characteristics for individual patients, thus contributing to determining the tumor immune phenotypes and guiding efficient clinical practice. Furthermore, the m^5^C-score might also be adopted to assess the clinicopathological characteristics of patients, like molecular subtypes, histological subtypes, tumor mutation burden, tumor inflammation stage, tumor differentiation degree, clinical stages, and genetic variation. This work elaborated the association of the m^5^C-score with the clinicopathological characteristics. Besides, the m^5^C-score also served as a biomarker to independently predict patient survival. The adjuvant chemotherapy efficacy and clinical anti-PD-1/PD-L1 immunotherapy response of patients were also predicted *via* the established m^5^C-score. Noteworthily, some new points were proposed in this study regarding cancer immunotherapy, which was that it was helpful to target the m^5^C regulators or the m^5^C phenotype-associated genes to alter m^5^C modification patterns, and to reverse the negative TME cell infiltration features, so as to develop new drug combinations and new immunotherapeutics. Results in this study shed new light on boosting immunotherapy response in patients, recognizing the diverse immune phenotypes of tumor and improving the individualized cancer immunotherapy.

To sum up, findings in the present study have illustrated the wide regulatory mechanisms of m^5^C methylation modification patterns in the TME. Heterogeneity in m^5^C modification patterns has been identified as a nonnegligible factor, which may induce TME complexity and heterogeneity. It is important to comprehensively evaluate the m^5^C modification patterns in individual tumors, so as to shed more light on TME cell-infiltrating features and to guide efficient immunotherapies.

## Data Availability Statement

The original contributions presented in the study are included in the article/**Supplementary Material**. Further inquiries can be directed to the corresponding author.

## Ethics Statement

The animal study was reviewed and approved by Institutional Review Board of The Second Affiliated Hospital of Anhui Medical University. Written informed consent was obtained from the individual(s) for the publication of any potentially identifiable images or data included in this article.

## Author Contributions

FL and QD conceived and designed the experiments. XP, SH, JZ, XZ, HC, and XL collected the data and performed the analysis. FL, QD, and XP participated in the discussion of the algorithm. FL, XP, SH, and JZ prepared and edited the manuscript. All authors contributed to the article and approved the submitted version.

## Funding

This research was supported by Clinical Research Cultivation Program of The Second Affiliated Hospital of Anhui Medical University (2020LCZD14) and Anhui Provincial Natural Science Foundation (2008085QH406).

## Conflict of Interest

The authors declare that the research was conducted in the absence of any commercial or financial relationships that could be construed as a potential conflict of interest.

## Publisher’s Note

All claims expressed in this article are solely those of the authors and do not necessarily represent those of their affiliated organizations, or those of the publisher, the editors and the reviewers. Any product that may be evaluated in this article, or claim that may be made by its manufacturer, is not guaranteed or endorsed by the publisher.

## Supplementary Material

Supplementary Figure 1 The work flow chart in the present work.

Supplementary Figure 2 Expression of 11 m^5^C regulators in normal samples and PTC samples in TCGA database.

Supplementary Figure 3 DEGs between different m^5^C modification patterns. **(A)** DEGs between m^5^C-cluster 1 and m^5^C-cluster 2. **(B)** DEGs between m^5^C-cluster 2 and m^5^C-cluster 3. **(C)** DEGs between m^5^C-cluster 1 and m^5^C-cluster 3.

Supplementary Figure 4 3 different m^5^C modification genome phenotypes were classified by unsupervised cluster analysis.

Supplementary Figure 5 The expression levels of 9 m^5^C regulators in 3 m^5^C gene-cluster subtypes.

Supplementary Figure 6 Enrichment analysis of DEGs between high and low m^5^C-score samples.

Supplementary Figure 7 The distribution of clinical features (gender, stage and age) of high and low m^5^C-score samples.

Supplementary Figure 8 Expression of 9 m^5^C regulators in high and low m^5^C-score samples.

Supplementary Figure 9 The correlation of m^5^C-score with TME. **(A)** The levels of infiltration of 21 immune cells in high and low m^5^C-score samples. **(B)** Levels of stromal scores, immune scores and ESTIMATE scores in high and low m^5^C-score samples.

Supplementary Figure 10 The expression levels of 16 immune checkpoints in high and low m^5^C-score samples.

Supplementary Table 1 Differentially expressed genes screened and analyzed by the R package limma.

Supplementary Table 2 Results of single factor survival analysis for the 49 genes.

Supplementary Table 3 Distribution of clinical features of the three subtypes.

Supplementary Table 4 Potential drugs for PTC screened and analyzed by l1000FWD tool.

Supplementary Table 5 Potential drugs for PTC screened and analyzed by CMAP tool.
