# Supplementary material for: m5C Regulator-Mediated Methylation Modification Patterns and Tumor Microenvironment Infiltration Characterization in Papillary Thyroid Carcinoma
Source: Front Oncol. 2021 Nov 3;11:729887. doi: 10.3389/fonc.2021.729887 (PMC8597900; doi:10.3389/fonc.2021.729887)
Supplement: Supplementary Figure 1 — The work flow chart in the present work. [file DataSheet_1.pdf]

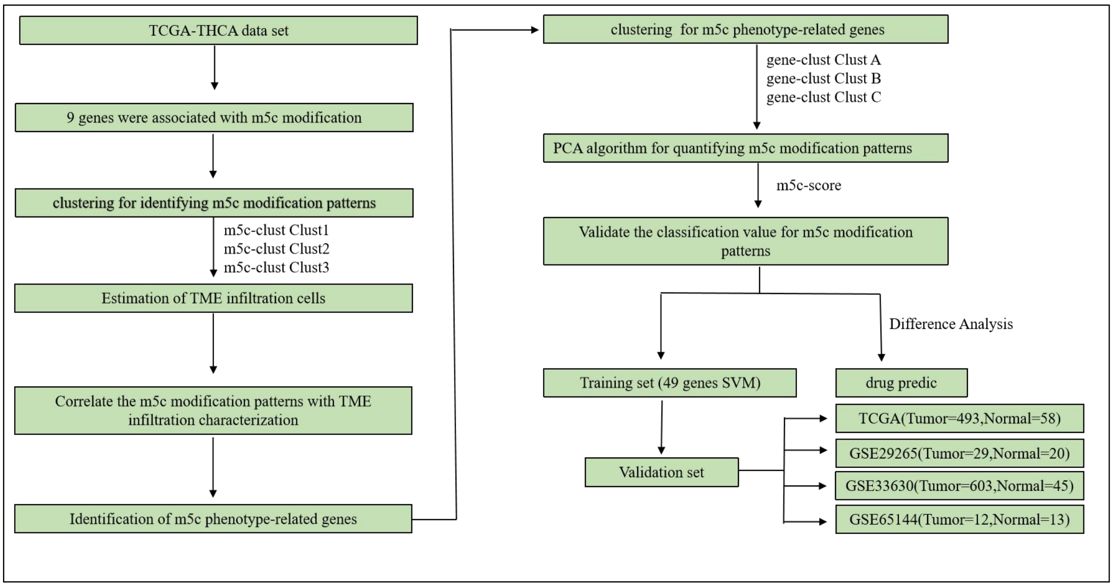

Supplementary Figure 1. The work flow chart in the present work.

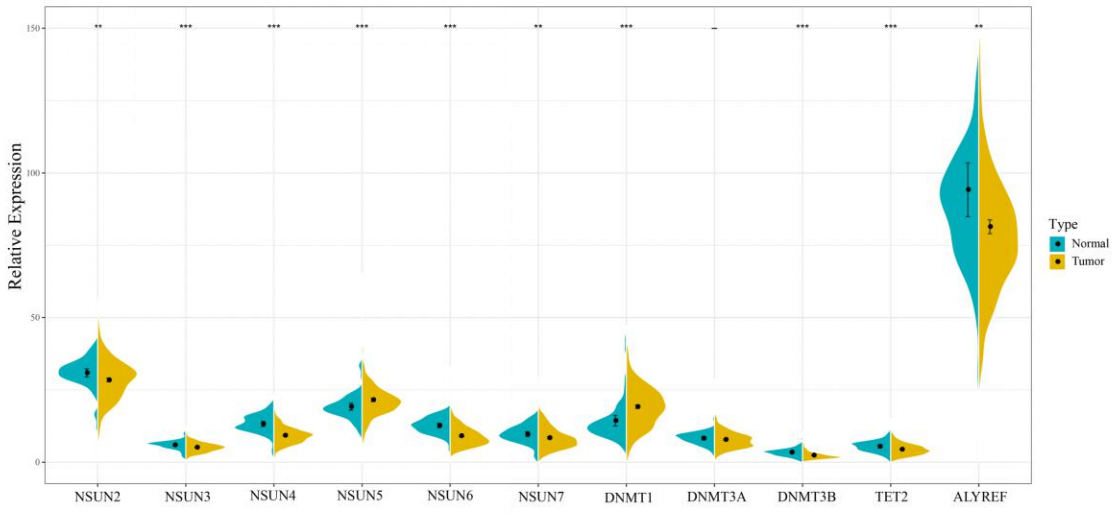

Supplementary Figure 2. Expression of 11 m^{5}C regulators in normal samples and PTC samples in TCGA database.

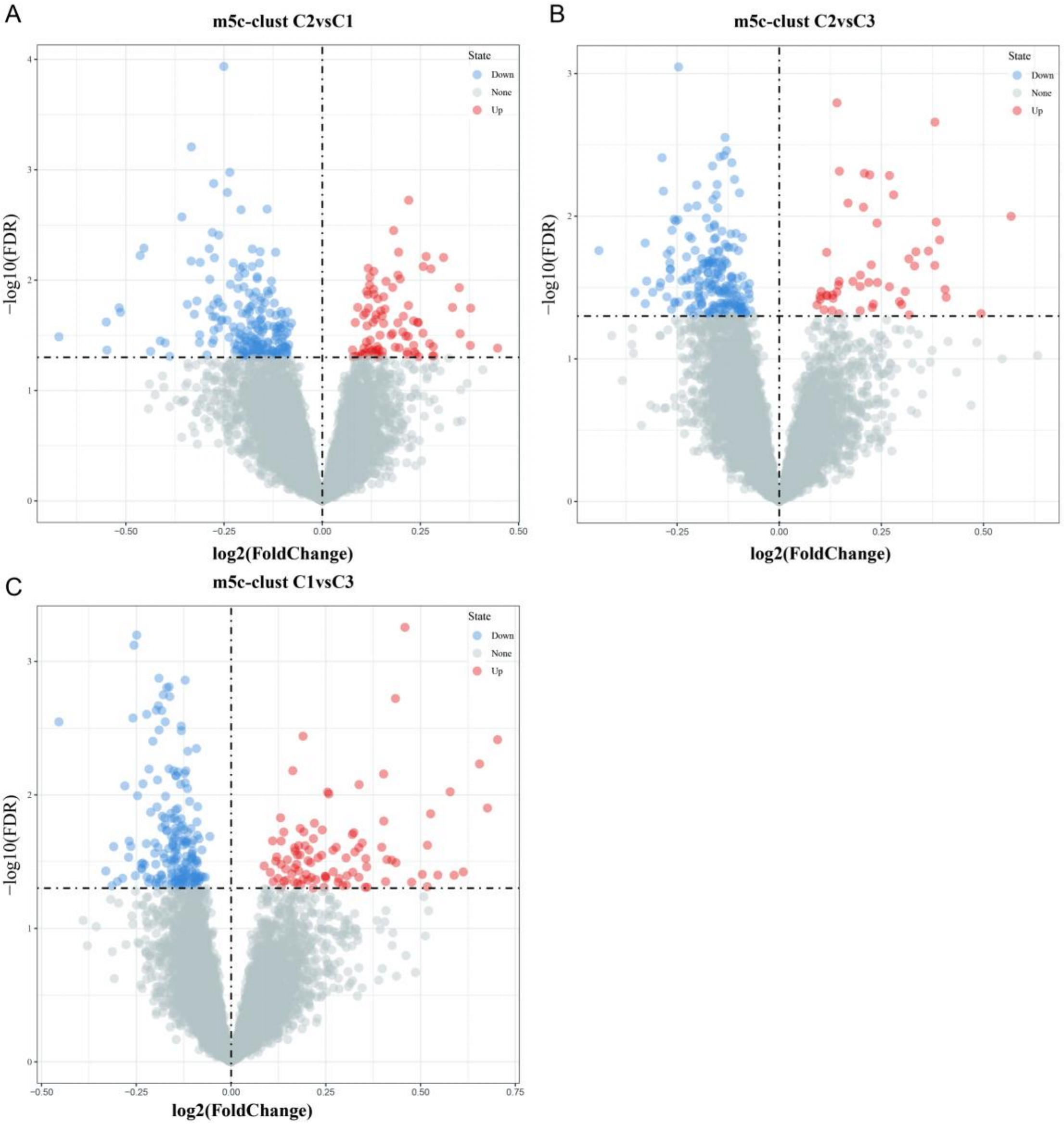

Supplementary Figure 3. DEGs betweendifferent m^{5}C modification patterns. (A) DEGs between m^{5}C-cluster 1 and m^{5}C-cluster 2. (B) DEGs between m^{5}C-cluster 2 and m^{5}C-cluster 3. (C) DEGs between m^{5}C-cluster 1 and m^{5}C-cluster 3.

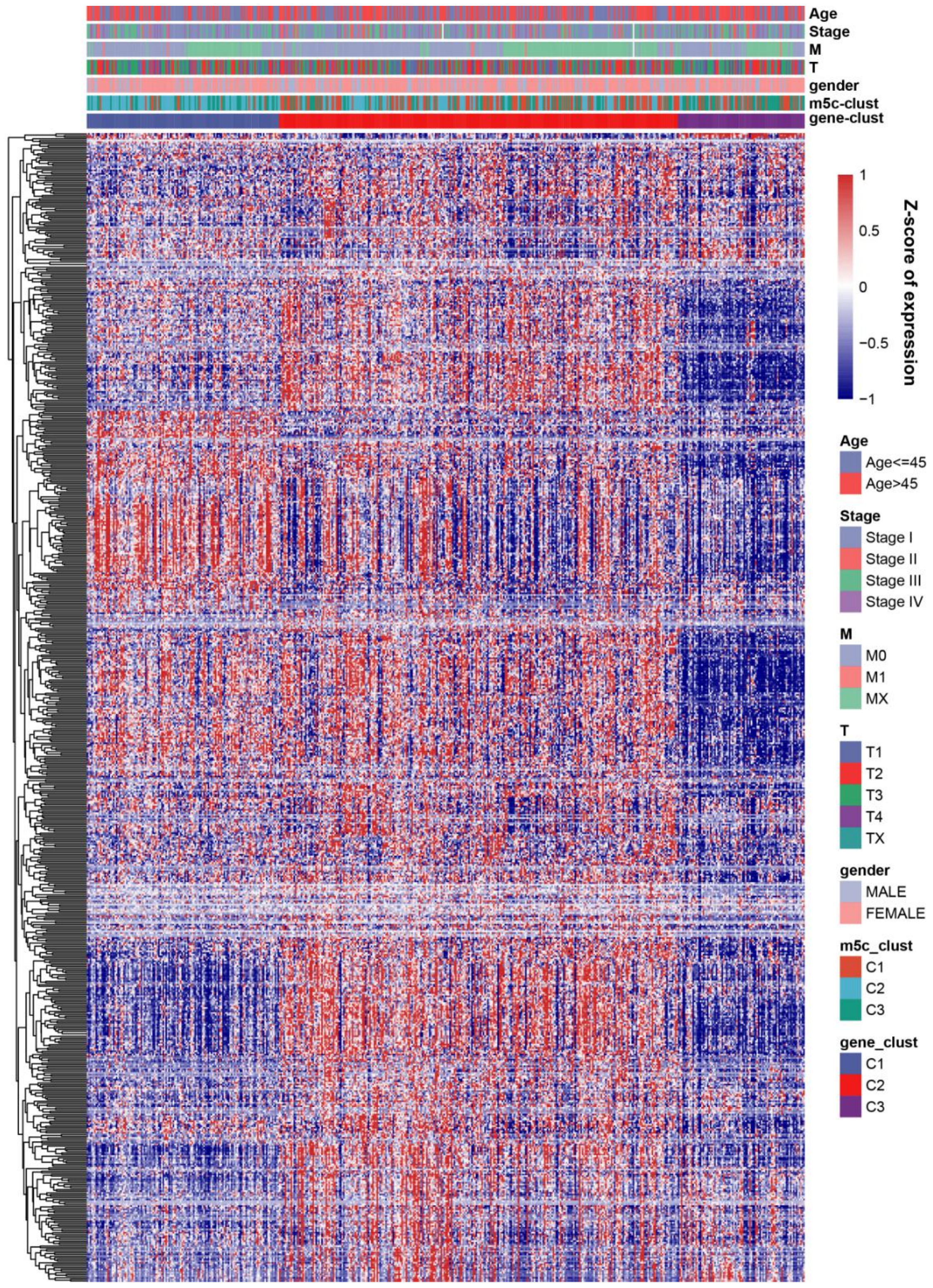

Supplementary Figure 4. 3 different m^5C modification genome phenotypes were classified by unsupervised cluster analysis.

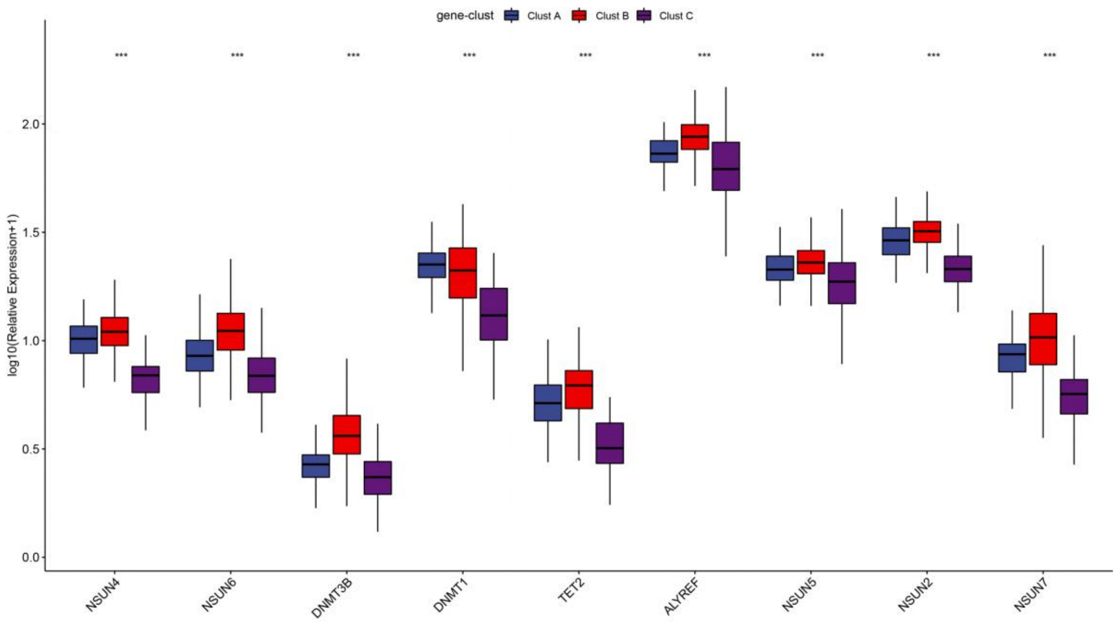

Supplementary Figure 5. The expression levels of 9 m^{5}C regulators in 3 m^{5}C gene-cluster subtypes.

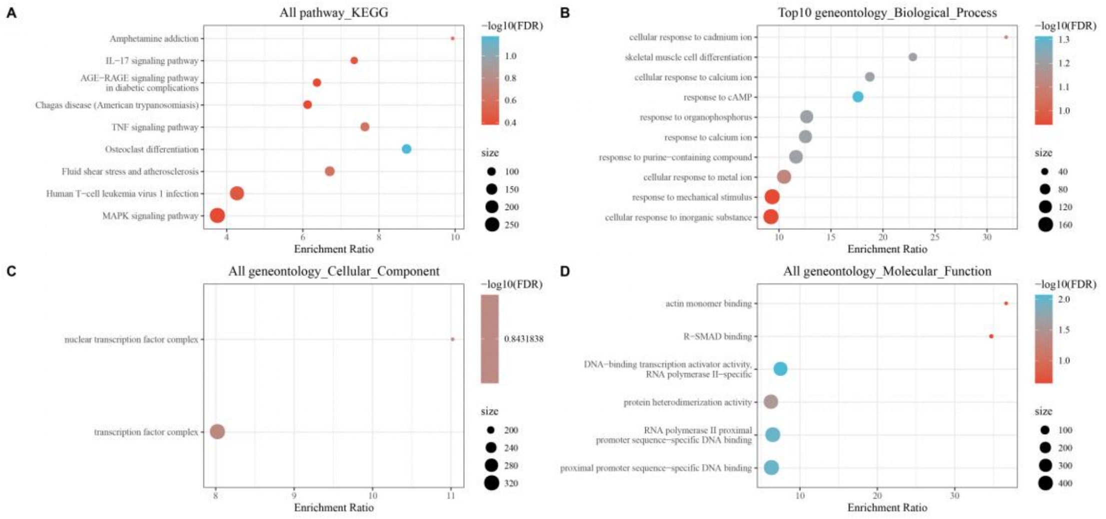

Supplementary Figure 6. Enrichment analysis of DEGs between high and low m^5C-score samples.

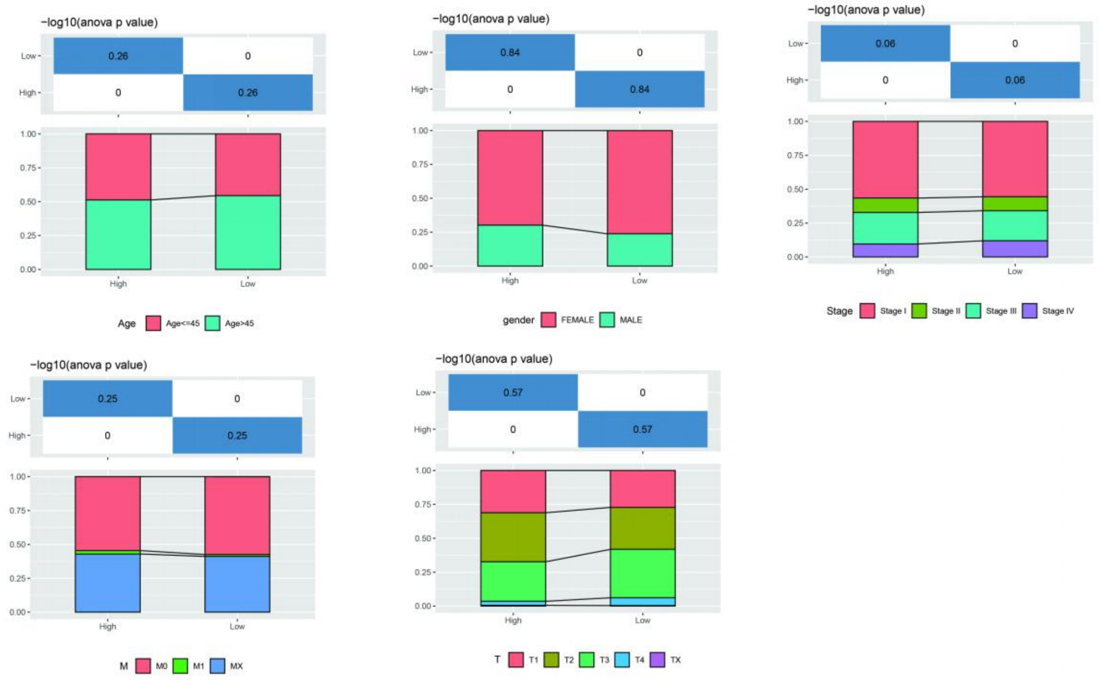

Supplementary Figure 7. The distribution of clinical features (gender, stage and age) of high and low m^{5}C-score samples.

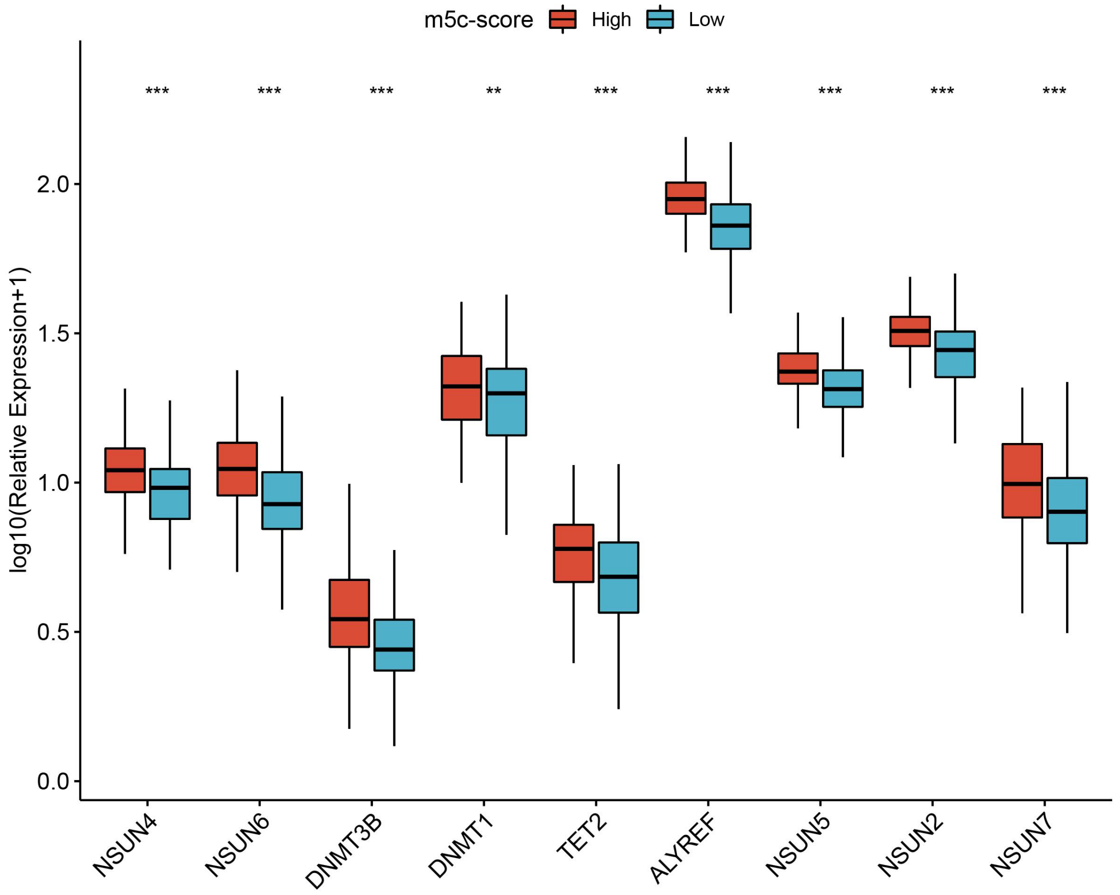

Supplementary Figure 8. Expression of 9 m^{5}C regulators in high and low m^{5}C-score samples.

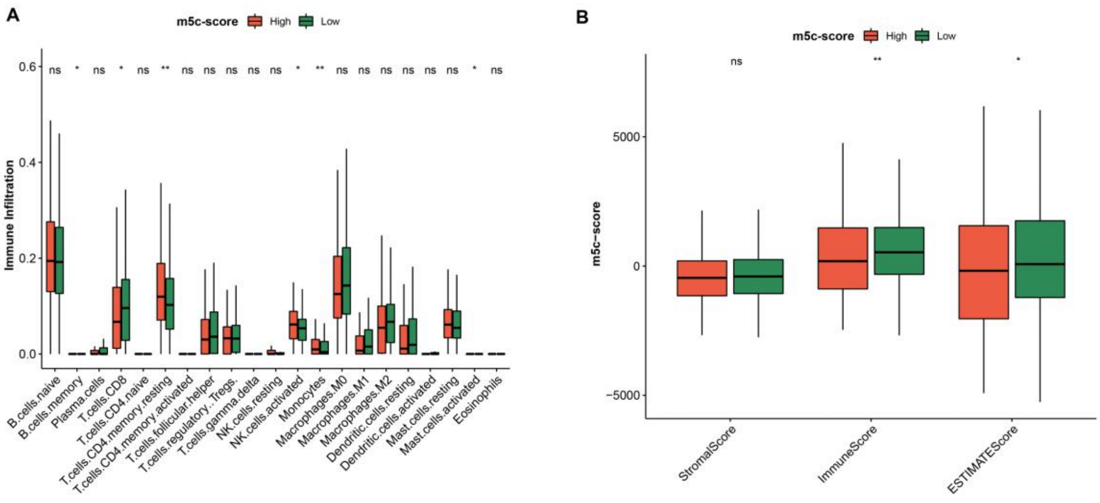

Supplementary Figure 9 The correlation of m^{5}C-score with TME. (A) The levels of infiltration of 21 immune cells in high and low m^{5}C-score samples. (B) Levels of stromal scores, immune scores and ESTIMATE scores in high and low m^{5}C-score samples.

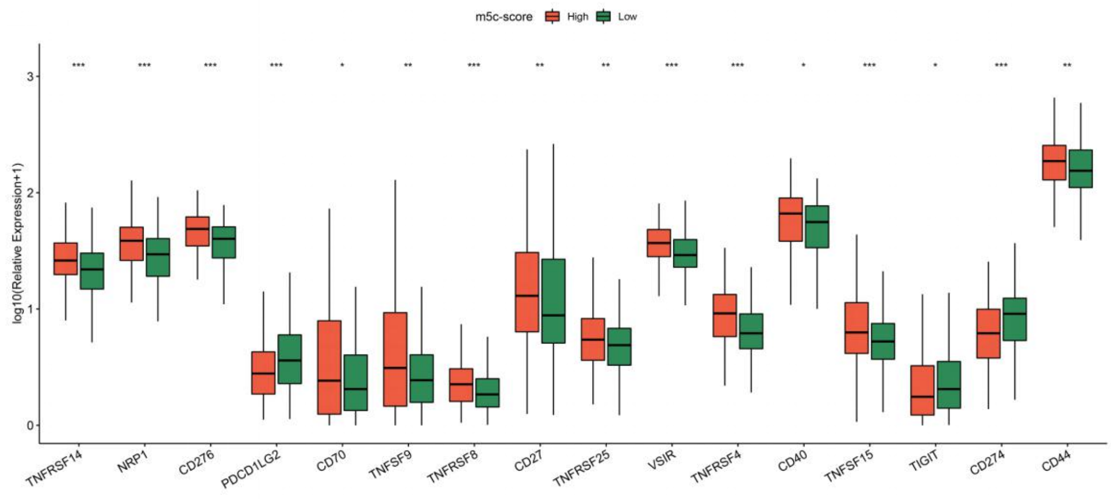

Supplementary Figure 10. The expression levels of 16 immune checkpoints in high and low m^5C-score samples.
